# Assessment of carotid atherosclerotic disease using three-dimensional cardiovascular magnetic resonance vessel wall imaging: comparison with digital subtraction angiography

**DOI:** 10.1186/s12968-020-0604-x

**Published:** 2020-03-05

**Authors:** Zhenjia Wang, Mi Lu, Wen Liu, Tiejin Zheng, Debiao Li, Wei Yu, Zhaoyang Fan

**Affiliations:** 1 Department of Radiology, Beijing Anzhen Hospital, Capital Medical University, No.2 Anzhen Road, Beijing, 100029 China; 2 Department of Radiology, Beijing Hospital of Traditional Chinese Medicine, Capital Medical University, No. 23, Back Road of Art Gallery, Beijing, 100010 China; 3 Department of Otolaryngology Head and Neck Surgery, Beijing Anzhen Hospital, Capital Medical University, No. 2 Anzhen Road, Beijing, 100029 China; 4 Department of Neurosurgery, Beijing Anzhen Hospital, Capital Medical University, No. 2 Anzhen Road, Beijing, 100029 China; 5 Biomedical Imaging Research Institute, Department of Biomedical Sciences, Cedars-Sinai Medical Center, 8700 Beverly Blvd., PACT 400, Los Angeles, CA 90048 USA; 6 Department of Bioengineering, University of California, Los Angeles, CA USA

**Keywords:** Vessel wall imaging, Carotid atherosclerotic disease, Magnetic resonance imaging, Validation

## Abstract

**Background:**

A three-dimensional (3D) cardiovascular magnetic resonance (CMR) vessel wall imaging (VWI) technique based on 3D T1 weighted (T1w) Sampling Perfection with Application-optimized Contrast using different flip angle Evolutions (SPACE) has recently been used as a promising CMR imaging modality for evaluating extra-cranial and intra-cranial vessel walls. However, this technique is yet to be validated against the current diagnostic imaging standard. We therefore aimed to evaluate the diagnostic performance of 3D CMR VWI in characterizing carotid disease using intra-arterial digital subtraction angiography (DSA) as a reference.

**Methods:**

Consecutive patients with at least unilateral > 50% carotid stenosis on ultrasound were scheduled to undergo interventional therapy were invited to participate. The following metrics were measured using 3D CMR VWI and DSA: lumen diameter of the common carotid artery (CCA) and segments C1–C7, stenosis diameter, reference diameter, lesion length, stenosis degree, and ulceration. We assessed the diagnostic sensitivity, specificity, accuracy, and receiver operating characteristic (ROC) curve of 3D CMR VWI, and used Cohen’s kappa, the intraclass correlation coefficient (ICC), and Bland-Altman analyses to assess the diagnostic agreement between 3D CMR VWI and DSA.

**Results:**

The ICC (all ICCs ≥0.96) and Bland-Altman plots indicated excellent inter-reader agreement in all individual morphologic measurements by 3D CMR VWI. Excellent agreement in all individual morphologic measurements were also found between 3D CMR VWI and DSA. In addition, 3D CMR VWI had high sensitivity (98.4, 97.4, 80.0, 100.0%), specificity (100.0, 94.5, 99.1, 98.0%), and Cohen’s kappa (0.99, 0.89, 0.84, 0.96) for detecting stenosis > 50%, stenosis > 70%, ulceration, and total occlusion, respectively, using DSA as the standard. The AUC of 3D CMR VWI for predicting stenosis > 50 and > 70% were 0.998 and 0.999, respectively.

**Conclusions:**

The 3D CMR VWI technique enables accurate diagnosis and luminal feature assessment of carotid artery atherosclerosis, suggesting that this imaging modality may be useful for routine imaging workups and provide comprehensive information for both the vessel wall and lumen.

## Background

Carotid atherosclerosis is increasingly recognized as the leading cause of ischemic stroke [1–3]. Previous studies have shown that accurate evaluation of the degree of carotid stenosis would be beneficial for predicting the risk of stroke [4–6]. Digital subtraction angiography (DSA) is still considered the clinical gold standard for assessing the severity of carotid atherosclerosis [7, 8]. However, several major limitations of DSA have impeded its widespread use in routine imaging workups, including invasiveness and potential complications, radiation exposure, high costs, and difficulty in visualizing lesions with positive remodeling [9, 10]. As a result, DSA is typically reserved for surgical interventions [7]. In contrast, ultrasound is a relatively cheap and portable method, and has been widely used for first-line diagnosis of atherosclerosis. However, ultrasound is susceptible to the level of experience of operators, and the use for patients with short, muscular necks is limited [11]. In contrast, high-resolution black-blood (BB) cardiovascular magnetic resonance (CMR) imaging can non-invasively visualize and quantify the vessel lumen and wall at a relatively low cost and without radiation exposure [12–16]. Hence, many groups have incorporated BB CMR in order to facilitate the diagnosis of carotid artery disease [17–21].

Recently, a three-dimensional (3D) CMR vessel wall imaging (VWI) technique based on 3D T1 weighted (T1w) Sampling Perfection with Application-optimized Contrast using different flip-angle Evolutions (SPACE) has become technically feasible and gained increasing attention for the diagnosis of atherosclerosis [22–26]. This technique is advantageous over conventional two-dimensional techniques, as it has sufficient BB effects, large spatial coverage, high isotropic resolution and signal-to-noise ratio (SNR), excellent scan efficiency, and remarkable cerebrospinal fluid (CSF) signal attenuation [23, 24, 27]. It therefore shows a unique capability for evaluating both lumen and wall features. Zhang et al. [27] demonstrated the reliability (excellent intra- and inter-observer agreement and scan-rescan reproducibility) of the 3D whole-brain intracranial VWI technique for quantifying intracranial vessel dimensions in 34 healthy subjects and 10 patients with known intracranial atherosclerotic disease. However, the diagnostic accuracy of 3D CMR VWI with respect to the luminal features involved in atherosclerosis has not been systematically validated against the clinical gold standard. Thus, we aimed to evaluate the performance of the 3D CMR VWI modality in diagnosing intra- and extra-cranial carotid atherosclerosis, using DSA as a reference.

## Methods

This study protocol was approved by the local Institutional Review Board of Beijing Anzhen Hospital (Capital Medical University, Beijing, China). Written informed consents were obtained from either the patients or their legal representatives prior to study entry.

### Participants

Between July 2015 and June 2018, the study prospectively recruited consecutive patients. The patients had been scheduled for DSA and interventions due to presentation with amaurosis fugax, transient ischemic attack, or suspected recent (< 14 days) cerebrovascular ischemia and the diagnosis of ≥50% carotid artery stenosis by ultrasound. All enrolled patients underwent 3D CMR VWI within 7 days before DSA. Patients were excluded if they had metallic or pacemaker implants, severe claustrophobia, renal dysfunction, or if they were allergic to iodinated contrast material. Of note, patients who were unable to tolerate the 20 min CMR imaging protocol were also excluded.

### MR imaging protocol

All examinations were performed on a 3-T whole-body CMR system (MAGNETOM Verio; Siemens AG Healthineers, Erlangen, Germany) using an 8-channel phased-array carotid surface coil (Shanghai Chenguang Medical technologies CO, LTD, Shanghai, China) and a 16-channel head coil (Siemens AG Healthineers). Patients were scanned in a supine, head-first position. After localization, 3D CMR VWI images were obtained using the following imaging parameters: sagittal orientation, echo time (TE)/ repetition time (TR) = 14/900 ms, field of view (FOV) = 180 × 200 × 120 mm^3^, spatial resolution = 0.63 × 0.63 × 0.63 mm^3^, matrix size = 284 × 320 × (208–240) with 7.7–25% partition oversampling, number of slices = 192, number of averages = 1, bandwidth = 488 Hz/pixel, parallel imaging (GRAPPA) factor = 2, and scan time = 7 min and 3 s. A trailing magnetization flip-down module was used to improve CSF signal attenuation [23]. The scan coverage included the C1-C7 segments of the bilateral internal carotid artery (ICA) and a distal portion of the common carotid artery (CCA).

### DSA imaging protocol

DSA was performed with a digital angiography system (Innova 3100; General Electric Healthcare, Waukesha, Wisconsin, USA) operated by an experienced neurointerventionalist. Seldinger’s technique was used to obtain patients’ images of the internal carotid artery (ICA), including the anterior-posterior and lateral projections. A total of 10 mL (rate, 4–5 mL/ sec) of contrast medium (Iopamidol 370, Bracco, Shanghai, China) was injected into the ICA. The parameters were as follows: matrix size = 1024 × 1024, spatial resolution = 2.75 LP/mm, and FOV = 30 cm.

### Image review

One neurointerventionalist with 17 years of experience (T.Z.) reviewed the DSA images and performed morphologic quantification at a workstation (AW4.7; General Electric Healthcare). The 3D CMR VWI images was reviewed and measured on a workstation (Leonardo, Siemens Healthineers, Erlangen, Germany) equipped with the multiplanar reconstruction (MPR) functionality.

Image quality (IQ) of CMR VWI was first assessed by an experienced reviewer (Y.W.) using a 4-point scale, where 1 = poor (insufficient for diagnosis, low SNR and obscured vessel wall or lumen boundaries), 2 = marginal (barely diagnosable, moderate SNR with a few motion or blood artifacts, distinguishable vessel wall and lumen boundary), 3 = good (appropriate for diagnosis, good SNR, distinguishable vessel wall, but partially obscured vessel lumen and wall boundaries), and 4 = excellent (sufficient for diagnosis; high SNR without artifacts, displaying vessel lumen boundary and wall clearly). Examples of IQ at different scale levels are provided in Online Figure 1. Images with IQ of 1 were excluded from further analyses [28, 29].

**Fig. 1:**
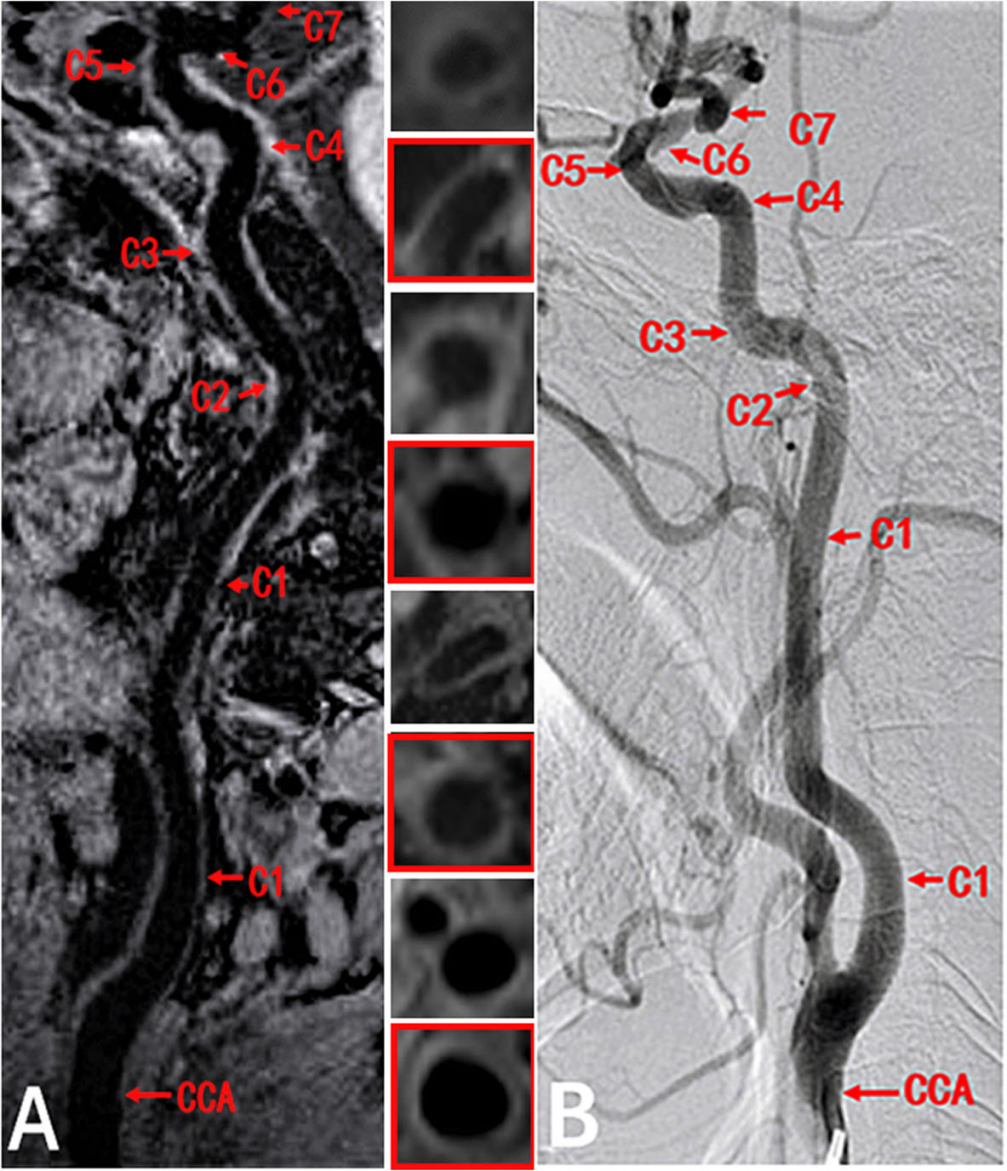
3D CMR VWI and DSA images of CCA. Both **a**, a sagittal 3D cardiovascular magnetic resonance (CMR) vessel wall imaging (VWI) image, and **b**, a lateral projection of a digital subtraction angiography (DSA) image, depict the lumen diameters of the common carotid artery (CCA) and C1-C7 segments in a 59-year-old man. The cross-sectional views at 8 locations are shown with numbered sub-windows, including CCA and internal carotid artery (ICA) (C1-C7). CCA, common carotid artery; 3D CMR VWI, three-dimensional cardiovascular magnetic resonance vessel wall imaging; DSA, digital subtraction angiography; ICA, internal carotid artery

Each ICA was divided into 7 segments (C1-C7) using the Bouthillier classification [30]. Data corresponding to each segment was measured in the 0.63-mm-thick cross-sections reformatted from 3D CMR VWI. A case example is provided in Fig. 1. The following variables were measured from the 3D CMR VWI and corresponding DSA images: 1) lumen diameter (i.e. the lumen diameter of the normal CCA and each ICA segment, stenosis diameter, and reference diameter); 2) stenosis degree (%), calculated according to the North American Symptomatic Carotid Endarterectomy Trial criteria [31]; 3) lesion length, measured as the maximum range of the lesion at the sagittal plane); and 4) presence of ulceration, defined as carotid plaque surface with fissuring or fibrous cap rupture. If a carotid artery had multiple stenoses, the narrowest one was evaluated. The severity of stenosis was subsequently classified according to the following criteria: no stenosis = 0%, mild stenosis = 0–29%, moderate stenosis = 30–69%, severe stenosis = 70–99%, and total occlusion = 100%. Two experienced radiologists (Z.W. and L.W.) blinded to the clinical information and DSA results, performed the above quantitatively analyses on the 3D CMR VWI images.

### Statistical analysis

Continuous variables are presented as mean ± standard deviation (SD), and categorical variables are reported as frequencies (percentages), as appropriate. Kolmogorov-Smirnov tests were used to access whether the measurements were normally distributed. Paired t-tests were used to test for the significant differences between readers or methods. The sensitivity, specificity, accuracy, positive predictive value, negative predictive value, and Cohen’s kappa with 95% confidence intervals of the 3D CMR VWI images were calculated for diagnosing total occlusion, carotid artery stenosis, and ulceration using DSA images as a reference. For continuous variables, the agreement between 3D CMR VWI and DSA, as well as the agreement between two CMR readers were assessed by using the intraclass correlation coefficient (ICC) and Bland-Altman analyses. The level of the agreement was categorized as follows: poor (ICC = 0–0.20); fair (ICC = 0.21–0.40); moderate (ICC = 0.41–0.60); good (ICC = 0.61–0.80); and excellent (ICC = 0.81–1.00) [32]. Receiver operating characteristic (ROC) curves were used to evaluate the performance of 3D CMR VWI at the DSA-stenosis cutoff values of 50 and 70%, respectively. All statistical analyses were performed using SPSS software, version 19.0 (Statistical Package for the Social Sciences, International Business Machines, Inc., Armonk, New York, USA). *P*-values < 0.05 were considered to indicate statistical significance.

## Results

### Image quality

**Fig. 2:**
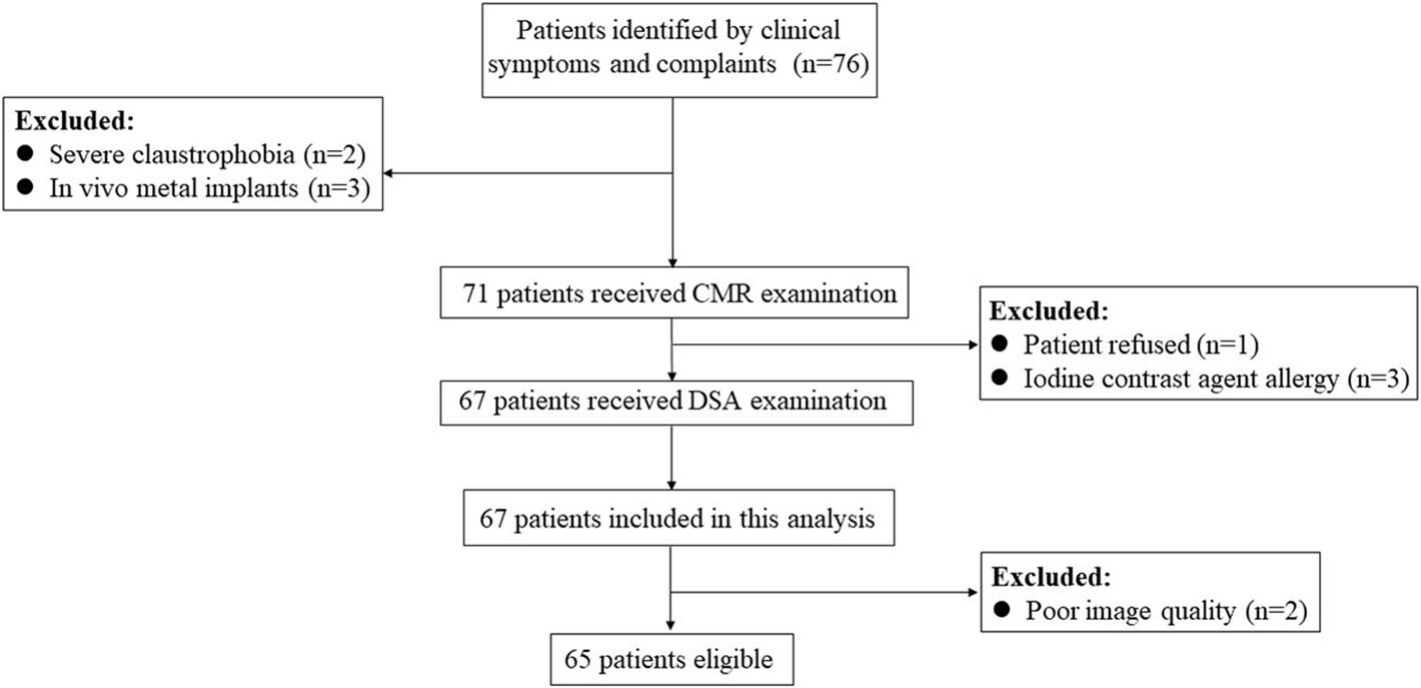
Flow chart of the study design. DSA, digital subtraction angiography; CMR, cardiovascular magnetic resonance

A total of 67 patients successfully underwent 3D CMR VWI and DSA. The overall image quality for 3D CMR VWI was 3.13 ± 0.73. Two patients were excluded from the study due to severe blood flow artifacts at the carotid bifurcation (*n* = 1) and severe motion artifacts (n = 1). In total, 65 patients were included in the final analyses. A detailed flow chart of the study design is presented in Fig. 2.

### Patient characteristics

Baseline demographics, medical history, and biochemical characteristics of the study population are shown in Table 1. The mean age of patients was 63.6 ± 5.5 years, and 52 (80.0%) subjects were male.

**Table 1:** Characteristics of the study’s cohort

Variables	Participants
Age, years	63.6 ± 8.56
Males, *n* (%)	52 (80.0%)
Hypertension, *n* (%)	44 (67.7%)
Hyperlipidemia, *n* (%)	55 (84.6%)
Diabetes mellitus, *n* (%)	35 (53.9%)
Coronary artery disease, *n* (%)	51 (78.5%)
Current smokers, *n* (%)	47 (72.3%)
Total cholesterol, mmol ∙L^−1^	3.76 ± 1.26
HDL cholesterol, mmol ∙L^− 1^	1.03 ± 0.39
LDL cholesterol, mmol ∙L^−1^	2.24 ± 0.98
Triglyceride, mmol∙L^−1^	1.36 ± 0.70
Homocysteine, umol∙L-1	14.38 ± 9.14
Glucose, mmol∙L-1	6.77 ± 2.02
LP(a), g∙L-1	0.24 ± 0.28
MTHFR677C-T	
CC, *n* (%)	6 (9.2%)
CT, *n* (%)	40 (61.5%)
TT, *n* (%)	19 (29.3%)

Data are presented as *n* (%) or mean ± standard deviation. HDL, high density lipoprotein, LDL, low density lipoprotein, LP(a), lipoprotein (a), MTHFR677C-T, methylenetetrahydrofolate reductase 677C and T

### Inter-reader agreement of the 3D CMR VWI images

**Fig. 3:**
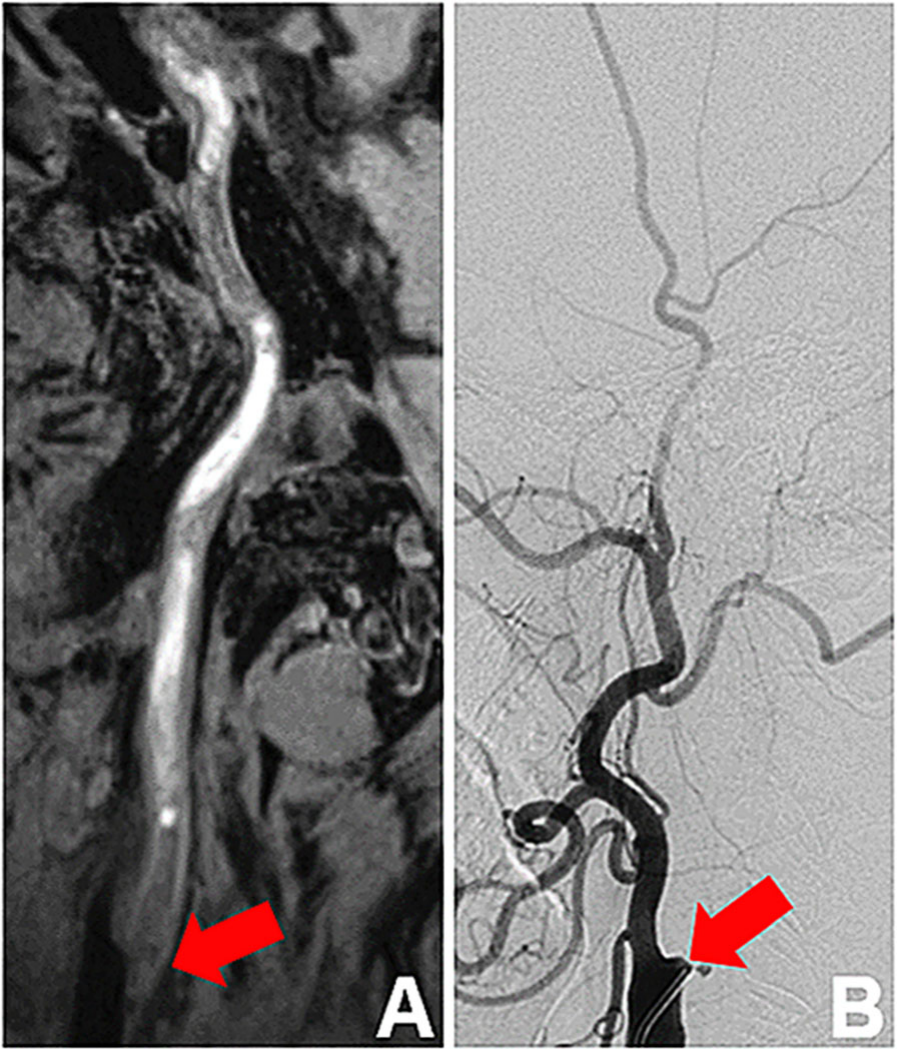
3D CMR VWI and DSA images of CCA of total occlusion. Both **a**, a sagittal 3D CMR VWI image, and **b**, a lateral projection of a DSA image depict total occlusion of the internal carotid artery (arrow) in a 67- year-old man. 3D CMR VWI, three-dimensional cardiovascular magnetic resonance vessel wall imaging; DSA, digital subtraction angiography

**Fig. 4:**
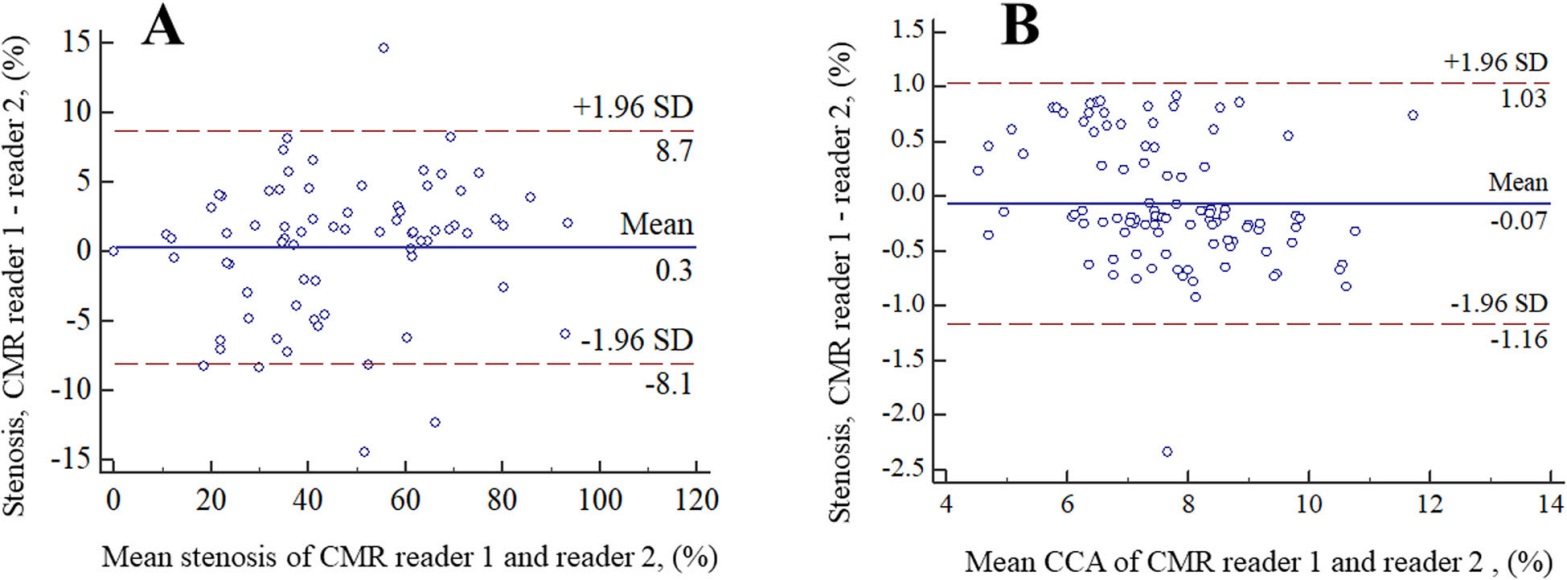
Bland-Altman plots of morphologic measurements between CMR reader 1 and reader 2 in all arteries without total occlusion. The solid lines represent the mean difference, and the dashed lines indicate the 95% limits of agreement. CCA, common carotid artery; SD, standard deviation; CMR, cardiovascular magnetic resonance

Of the 130 arteries, 31 total occlusions were identified by two CMR image readers with 100% inter-reader agreement. A case example of the total occlusion depicted by 3D CMR VWI is shown in Fig. 3a. The results of the morphologic measurements among the remaining 99 arteries and the arteries with stenosis > 50% identified by ultrasonography are summarized in Table 2. All ICCs of all morphologic indices were ≥ 0.96, indicating excellent inter-reader reproducibility. But there are significant differences among some metrics between CMR reader 1 and reader 2. The Bland-Altman plots of the stenosis degree and CCA diameter between CMR reader 1 and reader 2 in all of the arteries without total occlusion are shown in Fig. 4. The mean differences were 0.3% and − 0.07 mm, respectively.

**Table 2:** Inter-reader agreement of arteries without total occlusion on 3D CMR VWI images

Variables	All arteries (*n* = 99)						Arteries with stenosis > 50% of US (*n* = 48)					
	ICC	CMR reader 1	CMR reader 2	Mean Difference ± SE	*P*-value	Limits of Agreement	ICC	CMR reader 1	CMR reader 2	Mean Difference ± SE	*P* -value	Limits of Agreement
CCA, mm	0.96(0.94,0.97)	7.65 ± 1.34	7.72 ± 1.51	− 0.07 ± 0.06	0.244	−1.16,1.03	0.96(0.94,0.98)	7.48 ± 1.40	7.62 ± 1.66	−0.15 ± 0.08	0.087	−1.28,0.99
C1, mm	0.98(0.97,0.99)	4.65 ± 0.92	4.72 ± 0.90	−0.10 ± 0.03	0.001	−0.61,0.48	0.96(0.93,0.98)	4.49 ± 0.98	4.56 ± 0.90	−0.07 ± 0.05	0.164	−0.46,0.31
C2, mm	0.98(0.97,0.99)	4.15 ± 0.89	4.25 ± 0.88	−0.07 ± 0.04	0.050	−0.74,0.61	0.97(0.95,0.99)	4.03 ± 1.00	4.11 ± 1.00	−0.08 ± 0.05	0.081	−0.49,0.32
C3, mm	0.96(0.94,0.97)	4.13 ± 0.96	4.20 ± 0.93	−0.08 ± 0.04	0.024	−0.76,0.61	0.96(0.94,0.98)	3.93 ± 1.00	4.00 ± 0.99	−0.07 ± 0.05	0.067	−0.47,0.34
C4, mm	0.99(0.98,0.99)	3.61 ± 1.14	3.68 ± 1.10	−0.08 ± 0.03	0.017	−0.50,0.64	0.99(0.98,0.99)	3.43 ± 1.19	3.45 ± 1.16	−0.01 ± 0.04	0.700	−0.49,0.46
C5, mm	0.97(0.95,0.98)	3.90 ± 1.04	3.98 ± 1.01	−0.09 ± 0.04	0.036	−0.88,0.73	0.97(0.95,0.98)	3.77 ± 1.16	3.80 ± 1.11	−0.03 ± 0.06	0.618	−0.49,0.43
C6, mm	0.98(0.97,0.99)	3.04 ± 0.84	3.14 ± 0.84	−0.08 ± 0.02	0.002	−0.53,0.38	0.98(0.97,0.99)	2.95 ± 0.99	3.01 ± 1.01	−0.06 ± 0.04	0.101	−0.47,0.35
C7, mm	0.97(0.96,0.98)	2.87 ± 0.76	2.98 ± 0.75	−0.08 ± 0.03	0.012	−0.66,0.51	0.97(0.95,0.99)	2.76 ± 0.69	2.81 ± 0.68	−0.05 ± 0.03	0.122	−0.33,0.23
Stenosis (%)	0.99(0.99,0.99)	34.71 ± 27.77	34.42 ± 27.23	0.29 ± 0.43	0.501	−8.09,8.68	0.98(0.97,0.99)	57.50 ± 17.25	56.00 ± 17.36	1.50 ± 0.68	0.032	−10.71,7,71
Stenosis diameter, mm	0.99(0.99,0.99)	1.93 ± 1.65	1.97 ± 1.65	−0.04 ± 0.02	0.058	−0.43,0.35	0.99(0.98,0.99)	2.08 ± 0.98	2.17 ± 0.93	−0.09 ± 0.03	0.090	−0.53,0.35
Reference diameter, mm	0.99(0.99,0.99)	3.62 ± 2.47	3.67 ± 2.51	−0.05 ± 0.01	0.001	−0.34,0.24	0.99(0.99,0.99)	4.83 ± 1.21	4.89 ± 1.25	−0.06 ± 0.02	0.059	−0.39,0.28
Lesion length, mm	0.99(0.99,0.99)	8.59 ± 7.29	8.74 ± 7.37	−0.15 ± 0.03	<0.001	−0.83,0.53	0.99(0.99,0.99)	13.81 ± 6.23	14.02 ± 6.27	−0.20 ± 0.07	0.004	−1.13,0.72

Note: 31 arteries with total occlusion displayed on CMR images were excluded

Data are presented as mean ± standard deviation. Numbers in parentheses are 95% CI

3D CMR VWI, three-dimensional cardiovascular magnetic resonance vessel wall imaging; CCA, common carotid artery; CI, confidence interval; ICC, intraclass correlation coefficient; SD, standard deviation; SE, standard error; US, ultrasound

### Agreement between 3D CMR VWI and DSA

**Fig. 5:**
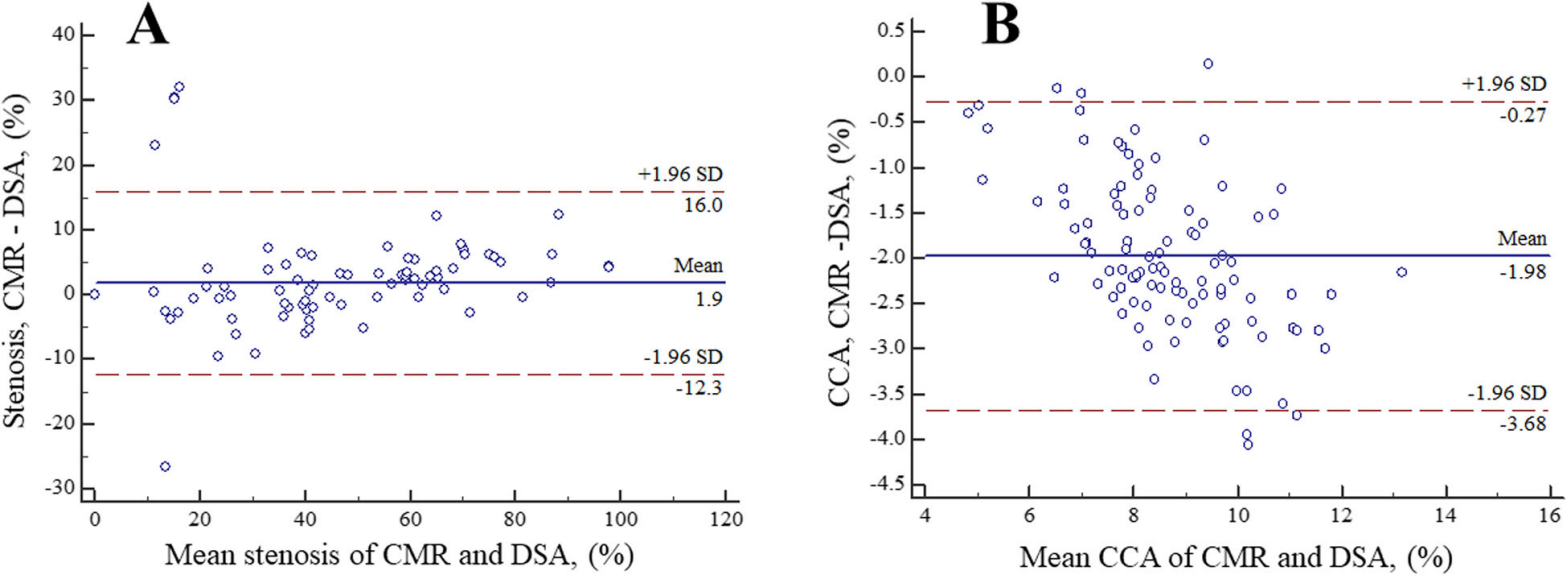
Bland-Altman plots of morphologic measurements as measured by CMR and DSA in all arteries without total occlusion. The solid lines represent the mean difference, and the dashed lines indicate the 95% limits of agreement. CCA, common carotid artery; DSA, digital subtraction angiography; CMR, cardiovascular magnetic resonance; SD, standard deviation

Of the 130 arteries, 29 total occlusions were identified by DSA. 3D CMR VWI revealed all the 29 total occlusions but mistook two pseudo-occlusive vessels for total occlusion. Thus, the sensitivity, specificity, accuracy, positive predictive value (PPV), negative predictive value (NPV), and Cohen’s kappa of 3D CMR VWI for diagnosing total occlusion were 100.0, 98.0, 98.5, 93.5, 100.0% and 0.96, respectively. A case example of the total occlusion assessed by DSA is shown in Fig. 3b. The agreements in the quantitative measurements between 3D CMR VWI reader 1 and DSA for the remaining 101 arteries and 50 arteries with stenosis > 50% identified by ultrasonography are summarized in Table 3. The ICC values indicated excellent agreement for all of the morphologic measurements between 3D CMR VWI and DSA (all ICCs ≥0.90) in the remaining 101 arteries. In the arteries with stenosis > 50% detected by ultrasonography, there was excellent agreement for all metrics (all ICCs ≥0.84) between 3D CMR VWI imaging and DSA. The Bland-Altman plots of the stenosis degree and CCA diameter between CMR and DSA in all arteries without total occlusion are presented in Fig. 5. The mean differences were 1.9% and − 1.98 mm, respectively.

**Table 3:** Summary of Agreement between DSA and 3D CMR VWI images

Variables	All arteries (*n* = 101)						Arteries with stenosis > 50% of US (*n* = 50)					
	ICC	CMR reader 1	DSA	Mean Difference ± SE	*P*-value	Limits of Agreement	ICC	CMR reader 1	DSA	Mean Difference ± SE	*P*-value	Limits of Agreement
CCA, mm	0.92(0.88,0.95)	7.67 ± 1.35	9.65 ± 1.82	− 1.98 ± 0.09	<0.001	−3.68, −0.27	0.91(0.85,0.95)	7.53 ± 1.41	9.44 ± 1.93	−1.91 ± 0.14	<0.001	−3.79, −0.04
C1, mm	0.98(0.94,0.97)	4.56 ± 1.12	5.12 ± 1.14	−0.55 ± 0.03	<0.001	−1.20,0.09	0.98(0.96,0.99)	4.31 ± 1.31	4.83 ± 1.29	−0.52 ± 0.05	<0.001	−1.27,0.23
C2, mm	0.97(0.95,0.98)	4.07 ± 1.06	4.63 ± 1.04	−0.56 ± 0.04	<0.001	−1.27,0.15	0.96(0.94,0.98)	3.86 ± 1.26	4.44 ± 1.19	−0.57 ± 0.06	<0.001	−1.47,0.33
C3, mm	0.95(0.92,0.96)	4.05 ± 1.12	4.55 ± 1.05	−0.51 ± 0.05	<0.001	−1.48,0.46	0.95(0.92,0.97)	3.78 ± 1.25	4.36 ± 1.13	−0.58 ± 0.07	<0.001	−1.57,0.41
C4, mm	0.97(0.96,0.98)	3.54 ± 1.24	3.98 ± 1.17	−0.44 ± 0.04	<0.001	−1.21,0.36	0.96(0.92,0.97)	3.29 ± 1.35	3.76 ± 1.22	−0.47 ± 0.08	<0.001	−1.52,0.58
C5, mm	0.93(0.89,0.95)	3.82 ± 1.17	4.34 ± 1.10	−0.51 ± 0.06	<0.001	−1.67,0.64	0.89(0.81,0.94)	3.62 ± 1.36	4.23 ± 1.23	−0.61 ± 0.11	<0.001	−2.18,0.96
C6, mm	0.96(0.95,0.98)	2.98 ± 0.94	3.39 ± 0.92	−0.41 ± 0.04	<0.001	−1.10,0.28	0.96(0.92,0.98)	2.83 ± 1.14	3.27 ± 1.06	−0.44 ± 0.06	<0.001	−1.33,0.45
C7, mm	0.92(0.88,0.95)	2.82 ± 0.85	3.23 ± 0.80	−0.41 ± 0.05	<0.001	−1.30,0.48	0.84(0.71,0.91)	2.65 ± 0.87	3.10 ± 0.73	−0.45 ± 0.09	<0.001	−1.64,0.73
Stenosis (%)	0.98(0.98,0.99)	36.00 ± 28.97	34.13 ± 27.89	1.87 ± 0.72	0.011	−12.28,16.02	0.99(0.98,0.99)	59.20 ± 18.87	56.12 ± 17.13	2.48 ± 0.56	<0.001	−5.28,10.25
Stenosis diameter, mm	0.93(0.89,0.95)	1.89 ± 1.65	2.10 ± 1.95	−0.22 ± 0.09	0.026	−2.09,1.66	0.99(0.99,0.99)	2.00 ± 1.04	2.35 ± 1.05	−0.35 ± 0.02	<0.001	−0.67, −0.03
Reference diameter, mm	0.93(0.90,0.96)	3.61 ± 2.47	3.96 ± 2.96	−0.35 ± 0.14	0.012	−3.0,2.3	0.97(0.95,0.98)	4.83 ± 1.21	5.40 ± 1.37	−0.57 ± 0.06	<0.001	−1.43,0.29
Lesion length, mm	0.96(0.95,0.98)	8.59 ± 7.29	6.08 ± 5.74	2.51 ± 0.25	<0.001	−2.3,7.3	0.98(0.97,0.99)	13.81 ± 6.23	10.35 ± 5.08	3.46 ± 0.20	<0.001	0.70,6.20

Note: 29 arteries with total occlusion displayed on DSA images were excluded. After measurement of the reference diameter and lesion length, 2 additional arteries with total occlusion displayed on CMR images were excluded

Comparison between CMR reader 2 and DSA was not shown because of a strong similarity with results for CMR reader 1

Data are presented as mean ± standard deviation. Numbers in parentheses are 95% CI

3D CMR VWI, three-dimensional cardiovascular magnetic resonance vessel wall imaging; CCA, common carotid artery; CI, confidence interval; DSA, digital subtraction angiography; ICC, intraclass correlation coefficient; SD, standard deviation; SE, standard error; US, ultrasound

### Diagnostic accuracy of 3D CMR VWI imaging

**Fig. 6:**
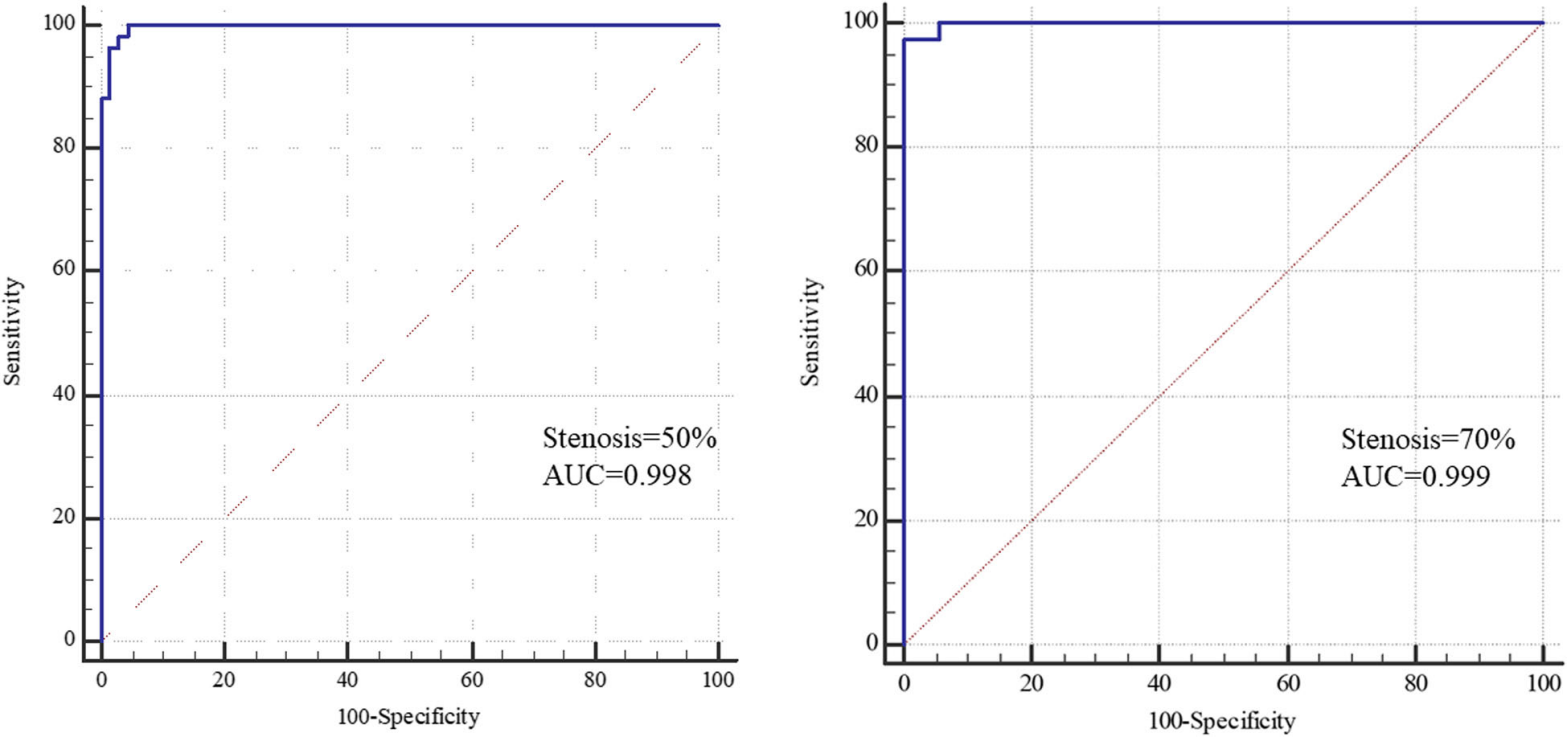
ROC curves of 3D CMR VWI according to various stenosis degree cutoff values. 3D CMR VWI, three-dimensional cardiovascular magnetic resonance vessel wall imaging; AUC: area under the curve; ROC: receiver operating characteristic

Using DSA as the reference, the sensitivity, specificity, positive predictive value, negative predictive value, accuracy, and Cohen’s kappa of 3D CMR VWI for detecting stenosis > 50%, stenosis > 70%, and total occlusion are presented in Table 4. Both CMR readers were able to identify the lesions. Of the 130 arteries, 20 (12.3%) arteries had ulcerations according to DSA, whereas CMR reader 1 detected 16 (80%) ulcerations on 3D CMR VWI. The sensitivity, specificity and Cohen’s kappa values were 80% (95% confidence interval [CI]: 62.5, 97.5%), 99.1% (95% CI: 97.3, 100%) and 0.84 (95% CI: 0.67, 1.00), respectively. In addition, 3D CMR VWI also had a high level of sensitivity (98.4, 97.4, 100.0%), specificity (100.0, 94.5, 98.0%), and Cohen’s kappa (0.99, 0.89, 0.96) for detecting stenosis > 50%, stenosis > 70%, and total occlusion, respectively, by MR reader 1. Figure 6 shows the ROC curve reflecting the diagnostic capability of 3D CMR VWI in the entire cohort when the threshold of stenosis was set at 50 and 70%, respectively.

**Table 4:** Sensitivity, Specificity, PPV, NPV, Accuracy and Cohen’s kappa of 3D CMR VWI images analysis using DSA as reference

Variables	Sensitivity		Specificity		PPV		NPV		Accuracy		Cohen’s kappa	
	CMR1	CMR2	CMR1	CMR2	CMR1	CMR2	CMR1	CMR2	CMR1	CMR2	CMR1	CMR2
Total Occlusion (*n* = 29)	100.0 (100.0,100.0)	100.0 (100.0,100.0)	98.0 (95.3,100.0)	98.0 (95.3,100.0)	93.5 (84.9,100.0)	93.5 (84.9,100.0)	100.0 (100.0,100.0)	100.0 (100.0,100.0)	98.5 (96.3,100.0)	98.5 (96.3,100.0)	0.96 (0.79,1.00)	0.96 (0.79,1.00)
Ulcer (*n* = 20)	80.0 (62.5,97.5)	85.0 (69.4,100.0)	99.1 (97.3,100.0)	98.2 (95.7,100.0)	94.1 (82.9,100)	89.5 (75.7,100.0)	96.4 (93.1,99.9)	97.3 (94.3,100.0)	96.2 (92.8,99.5)	96.2 (92.8,99.5)	0.84 (0.67,1.00)	0.85 (0.68,1.00)
Stenosis (%)												
>50% (*n* = 60)	98.4 (95.2,100.0)	96.8 (92.4,100.0)	100.0 (100.0,100.0)	98.5 (95.7,100.0)	100.0 (100.0,100.0)	98.4 (95.2,100.0)	98.6 (95.8,100.0)	97.1 (93.1,100.0)	99.2 (97.7,100.0)	97.7 (95.1,100.0)	0.99 (0.81,1.00)	0.95 (0.78,1.00)
>70% (*n* = 38)	97.4 (92.5,100.0)	97.4 (92.5,100.0)	94.5 (89.8,99.2)	97.8 (94.8,100.0)	88.4 (78.8,98.0)	95.0 (88.2,100.0)	98.9 (96.6,100.0)	98.9 (96.7,100.0)	95.4 (91.8,99.0)	97.7 (95.1,100.0)	0.89 (0.72,1.00)	0.95 (0.77,1.00)

All data, except for Cohen’s kappa, are percentages. Numbers in parentheses are 95% CI

3D CMR VWI, cardiovascular magnetic resonance three-dimensional vessel wall imaging; DSA, digital subtraction angiography; NPV, negative predictive value; PPV, positive predictive value

## Discussion

This study confirms the accuracy of the 3D CMR VWI technique based on the 3D T1w SPACE sequence for the diagnosis of carotid atherosclerosis, particularly the assessment of various luminal features, in symptomatic patients with ≥50% carotid artery stenosis. We found that the 3D CMR VWI technique is highly consistent with DSA when measuring the lumen diameter of the CCA and segments C1-C7 segments, as well as the stenosis diameter, stenosis degree, and reference diameter. Additionally, this technique showed excellent accuracy for diagnosing total occlusion, ulcerative lesions, and moderate to severe stenosis. Hence, our results justify the increasing use of 3D CMR VWI as a reliable, accurate, and non-invasive imaging modality for diagnosing carotid artery disease.

Analysis of inter-observer reproducibility is an essential requirement for validation of an imaging modality. Zhang et al. [27] previously performed a comprehensive reliability analysis for the 3D whole-brain CMR VWI technique. However, this study was limited by the small sample size of clinical patients. In our study, two experienced radiologists evaluated the intra- and extra-cranial carotid walls using 3D CMR VWI in 65 patients. Our results showed excellent inter-observer reproducibility for measurement of the lumen diameter of the CCA and segments C1-C7 of the carotid artery, stenosis diameter, stenosis degree, and reference diameter. There were systematic differences in diameter measurements, particularly at large-size vessel segments, between reader 1 and 2. This may be due to the different measurement habits among readers and non-circular lumen shapes of the large-size segments. Nevertheless, these differences were all submillimeters and not clinically relevant for diagnosis. Thus, 3D CMR VWI is considered to be a reliable imaging modality for the quantification of luminal morphologic features.

In this study, we evaluated the agreement between 3D CMR VWI and DSA. The morphologic measurements, including the lumen diameter of the CCA and segments C1-C7, and the stenosis diameter, stenosis degree, reference diameter, and lesion length, as measured by 3D CMR VWI, displayed excellent accordance with those measured by DSA (ICC > 0.84). One possible reason for the excellent performance of the 3D CMR VWI technique is that a non-selective excitation radio-frequency pulse has been used to shorten the echo time, which helps improve the SNR of images [23, 24, 27]. Additionally, 3D CMR VWI provided a high isotropic resolution of 0.63 × 0.63 × 0.63 mm^3^, and achieves good flexibility with regard to image visualization, which thereby enables the visualization of plaques located in the tortuous and deep-seated intracranial carotid. Furthermore, dedicated CSF signal suppression is implemented in this technique, which can help to visualize the outer boundaries of the vessel wall [23, 27].

However, our study showed some systematic differences between CMR VWI and DSA. Firstly, the lumen diameter tended to be underestimated by VWI at larger sized vessel segments. This discrepancy might be caused by the underlying differences in image acquisition and measurement. Specifically, DSA is a projection-based imaging modality and the distance between two parallel vessel boundaries from the imaging perspective is inherently the largest possible diameter of the vessel from that perspective. However, diameter measurement on VWI is performed from reformatted cross-sectional images and derived by averaging two perpendicular hand-drawn straight-line distances. Either of these lines is necessarily aligned with the DSA perspective, and more importantly, each distance may not represent the true diameter at that angle if the straight line misses the lumen center, thus underestimating the diameter. The averaging procedure would further make the measured diameter smaller, particularly at larger vessel segment that are not necessarily round. Secondly, the lesion lengths measured by 3D CMR VWI are about 3 mm longer than those measured by DSA in arteries with > 50% stenosis. The potential reason may be the distal portion of a wedge-shaped plaque being regarded as the reference on DSA [33, 34].

In our study, 3D CMR VWI showed a poor ability to identify pseudo-occlusion and small ulcerations (width < 1.5 mm), compared with DSA. Mistaking pseudo-occlusion for total occlusion on 3D CMR VWI might be explained by the fact that DSA has much better spatial and temporal resolution than 3D CMR VWI. This is in line with the findings of a previous study [20]. Missing small ulceration is attributed not only to the poorer spatial resolution but also to the existence of tiny calcification in the surface of the lumen [12, 20]. Therefore, we should pay more attention to the irregular calcification on the 3D CMR VWI images so as to detect ulceration properly. A previous study showed that bright-blood sequences are highly accurate in the detection of ulcerations because of a good depiction of the luminal boundary. Therefore, future studies which examine the combination of bright-blood sequences with 3D T1w SPACE sequences for the comprehensive diagnosis of ulceration are warranted [35].

Zhao et al. [20] performed a study similar to the current one, and showed that 3D BB MR imaging could accurately diagnose patients with moderate to severe carotid artery stenosis, which is consistent with the findings of our study. One potential limitation of their study is that they only assessed the consistency and accuracy of 3D BB MR in carotid lesions with > 50% stenosis. However, an increasing number of studies have found that carotid lesions with low-grade stenosis may also lead to stroke [4–6]. Moreover, cerebral angiography is often required before carotid artery stent implantation in order to observe the intracranial Willis ring opening and cerebral blood flow perfusion. Therefore, our study performed a comprehensive analysis of the consistency and accuracy of 3D CMR VWI in lesions with both less than and greater than 50% stenosis. We showed excellent consistency for measurement of lumen diameter and stenosis in all arteries between 3D CMR VWI and DSA. However, CMR VWI may be less accurate for more severe lesions. Briefly, there are two potential reasons why this may be the case. First, 3D CMR VWI has poorer spatial resolution than DSA, meaning that the measurement errors induced by partial volume effect become more evident in cases of severe stenoses. Second, blood signal suppression may become more challenging as the severity of stenosis increases, resulting in residual flow artifacts particularly at juxta-luminal locations. Another limitation of their study was that they only assessed lesions located at the level of carotid bifurcation and not segments C2-C7, whilst carotid atherosclerosis is a pan-vascular disease involving intra- and extra- cranial arteries. 3D CMR VWI images can cover the whole internal carotid artery within 7 min. Assessment of lesions within a large FOV is useful for the selection of stent size and the formulation of surgical procedures [36].

There were several limitations in this study. Firstly, measurements of 3D CMR VWI were acquired using MPR. Therefore, discrepancy from mismatched slices of the carotid artery lesions between 3D CMR VWI and DSA may have compromised the agreement analysis. Secondly, 3D CMR VWI was still susceptible to motion and residual blood flow artifacts which occurred at certain anatomical locations, including carotid bifurcations and petrous and lacerum segments of ICAs, which can interfere with the correct assessment of the condition [22]. For example, 3D CMR VWI reader 1 misdiagnosed 6 artifacts as stenosis in our study. Thirdly, CMR angiography was not included in our imaging protocol. There were two main reasons for this design. First, our study primarily aimed to evaluate the diagnostic performance of 3D CMR VWI in characterizing carotid disease by comparison with the clinical gold standard (DSA), but not to focus on the agreement analysis between 3D CMR VWI and MRA. Second, carotid CMR angiography acquired by time-of-flight (TOF) or contrast-enhanced gradient recalled echo (GRE) typically has a much lower spatial resolution than 3D CMR VWI (isotropic 0.63 mm).

## Conclusion

3D CMR VWI can be used to accurately assess carotid atherosclerosis with high spatial resolution and large anatomical coverage in a single scan, suggesting that the imaging modality may serve in routine imaging workup with comprehensive information for both the vessel wall and lumen.

## Supplementary information

**Additional file 1:** Online Figure 1. Example images of image quality at different scale levels. A, image quality score = 4; B, image quality score = 3; C, image quality score = 2; D, image quality score = 1.

## Data Availability

The datasets used and/or analyzed during the current study are available from the corresponding author on reasonable request.

## Abbreviations

AUC: Area under the curve
3D: Three-dimensional
BB: Black blood
CCA: Common carotid artery
CI: Confidence interval
CMR: Cardiovascular magnetic resonance
CSF: Cerebral spinal fluid
DSA: Digital subtraction angiography
FOV: Field-of-view
GRE: Gradient recalled echo
HDL: High density lipoprotein
ICA: Internal carotid artery
ICC: Intraclass correlation coefficient
IQ: Image quality
LDL: Low density lipoprotein
LP(a): Lipoprotein (a)
MPR: Multiplanar reconstruction
MTHFR677C-T: Methylenetetrahydrofolate reductase 677C-T
NPV: Negative predictive value
PPV: Positive predictive value
ROC: Receiver operator characteristics
SD: Standard deviation
SE: Standard error
SNR: Signal-to-noise ratio
SPACE: Sampling Perfection with Application-optimized Contrast using different flip angle Evolutions
T1w: T1 weighted
TE: Echo time
TOF: Time-of-flight
TR: Repetition time
US: Ultrasound
VWI: Vessel wall imaging
