# Supplementary figures and images for: Assessment of carotid atherosclerotic disease using three-dimensional cardiovascular magnetic resonance vessel wall imaging: comparison with digital subtraction angiography
Source: J Cardiovasc Magn Reson. 2020 Mar 5;22:18. doi: 10.1186/s12968-020-0604-x (PMC7057661; doi:10.1186/s12968-020-0604-x)

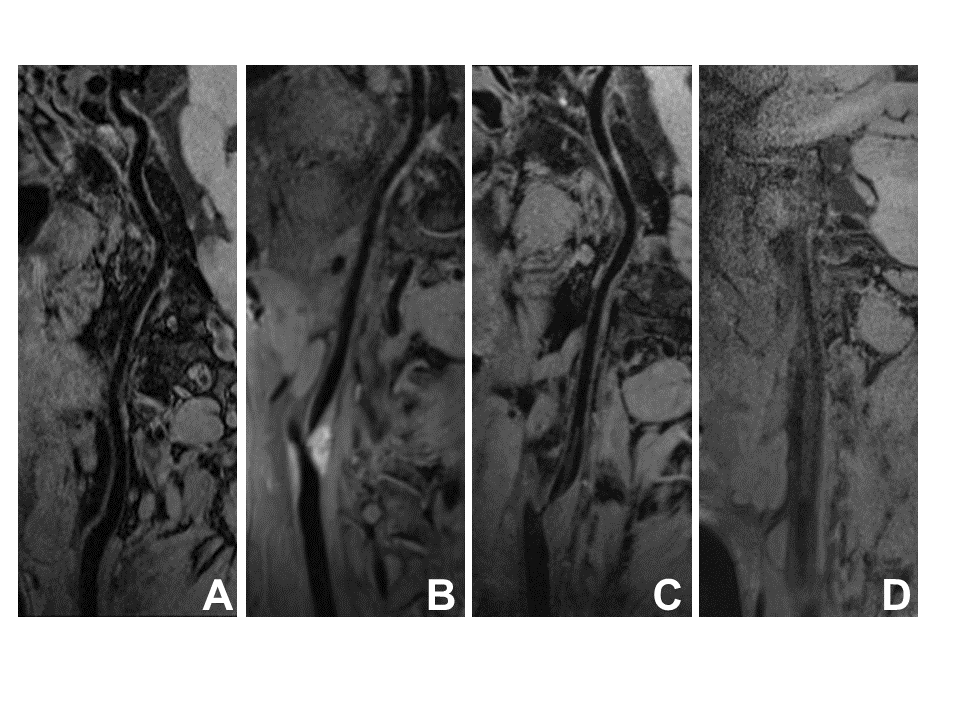

Supplement: Supplementary file 1 — Additional file 1: Online Figure 1. Example images of image quality at different scale levels. A, image quality score = 4; B, image quality score = 3; C, image quality score = 2; D, image quality score = 1. [file 12968_2020_604_MOESM1_ESM.png]
